# A Systematic Review on Combined [^18^F]FDG and ^68^Ga-SSA PET/CT in Pulmonary Carcinoid

**DOI:** 10.3390/jcm12113719

**Published:** 2023-05-28

**Authors:** Daniela Prosperi, Luciano Carideo, Vincenzo Marcello Russo, Rosaria Meucci, Giuseppe Campagna, Secondo Lastoria, Alberto Signore

**Affiliations:** 1 Nuclear Medicine Unit, University Hospital Sant’Andrea, Via di Grottarossa 1035, 00189 Rome, Italy; 2 Nuclear Medicine Unit, IRCCS National Cancer Institute, Fondazione Senatore G. Pascale, 80127 Naples, Italy; 3 Nuclear Medicine Unit, Department of Medical-Surgical Sciences and of Translational Medicine, Faculty of Medicine and Psychology, “Sapienza” University, 00184 Rome, Italyalberto.signore@uniroma1.it (A.S.); 4 U.O.C. Diagnostic Imaging, PTV Policlinico “Tor Vergata” University, Viale Oxford 81, 00133 Rome, Italy

**Keywords:** pulmonary carcinoid, typical carcinoid, atypical carcinoid, [^18^F]FDG, ^68^Ga-SSA

## Abstract

Pulmonary carcinoids (PCs) are part of a spectrum of well-differentiated neuroendocrine neoplasms (NENs) and are classified as typical carcinoid (TC) and atypical carcinoid (AC). TC differ from AC not only for its histopathological features but also for its “functional imaging pattern” and prognosis. ACs are more undifferentiated and characterized by higher aggressiveness. Positron emission tomography/computed tomography (PET/CT) with somatostatin analogs (SSA) labeled with Gallium-68 (^68^Ga-DOTA-TOC, ^68^Ga-DOTA-NOC, ^68^Ga-DOTA-TATE) has widely replaced conventional imaging with gamma camera using ^111^In- or ^99m^Tc-labelled compounds and represents now the gold standard for diagnosis and management of NENs. In this setting, as already described for gastro-entero-pancreatic NENs, ^18^F-Fluorodeoxiglucose ([^18^F]FDG) in addition to ^68^Ga-SSA can play an important role in clinical practice, particularly for ACs that show a more aggressive behavior compared to TCs. The aim of this systematic review is to analyze all original studies collected from the PubMed and Scopus databases regarding PCs in which both ^68^Ga-SSA PET/CT and [^18^F]FDG PET/CT were performed in order to evaluate the clinical impact of each imaging modality. The following keywords were used for the research: “^18^F, ^68^Ga and (bronchial carcinoid or carcinoid lung)”. A total of 57 papers were found, of which 17 were duplicates, 8 were reviews, 10 were case reports, and 1 was an editorial. Of the remaining 21 papers, 12 were ineligible because they did not focus on PC or did not compare ^68^Ga-SSA and [^18^F]FDG. We finally retrieved and analyzed nine papers (245 patients with TCs and 110 patients with ACs), and the results highlight the importance of the combined use of ^68^Ga-SSA and [^18^F]FDG PET/CT for the correct management of these neoplasms.

## 1. Introduction

Pulmonary carcinoids (PCs) are rare malignant neoplasms being part of well-differentiated pulmonary neuroendocrine neoplasia (NEN) arising from stem cells of the bronchial epithelium known as Kulchitsky cells [1]. Even though in the majority of cases NENs arise at the gastroenteropancreatic level, PCs are the second most frequent location and represent about 25% of primary lung neoplasm with an approximate annual incidence of 2.3–2.8 cases per million [2]. No age and sex prevalence have been reported, and neither external environmental toxin or other risk factors have been detected [3]. PCs can be classified into two categories according to histological features: typical carcinoids (TCs) are well-differentiated, low-grade (less than two mitoses per 2 mm^2^), absence of necrosis and less aggressive; by contrast, atypical carcinoids (ACs) are poorly differentiated, intermediate-grade (more than two mitoses per 2 mm^2^) and necrosis is potentially present. These different histological patterns are strictly correlated with prognosis [4]. Typical carcinoids (TCs) have a good prognosis as a result of less aggressive behavior, while atypical carcinoids (ACs) are more aggressive at diagnosis with a higher risk of lymph node and distant metastases [5].

Based on histological features, approximately 60 to 80% of PCs share the unique feature of over-expressing somatostatin receptors (SSTR), particularly in lower grade lesions characterized by low Ki-67 proliferation index [6]. During the gradual progression of carcinogenesis NENs may show a dedifferentiation with development of more aggressive tumor cell clones. This behavior, results in a loss of somatostatin receptors and utilization of glucose metabolism that correlates with a worse prognosis. Although final diagnosis of PCs is made through histopathologic examination, nonetheless, non-invasive imaging methods can provide information aiding in the prediction of the histopathologic subtype of carcinoid and reliably guide the patient management and invasiveness of surgery [7]. In this context, nuclear medicine plays a crucial role in the characterization of these neoplasms.

In this systematic review we analyze all papers published on both TCs and ACs studied by both [^18^F]FDG and ^68^Ga-SSA PET/CT, focusing on the impact of their combined use in clinical practice.

## 2. Materials and Methods

### 2.1. Search Strategy

We followed the Preferred Reporting Items for Systematic Reviews and Meta-Analysis (PRISMA) method [8], and the search was carried out in the following databases: PubMed (Medline) and SCOPUS. The search terms used were as index terms or free-text words, and the complete strategy was as follows: “^18^F, ^68^Ga AND (bronchial carcinoid or carcinoid lung”.

### 2.2. Data Collection

Basic information about studies (authors, publication year, country of study, study design, patients’ characteristics, and technical aspects) was collected.

Two researchers independently reviewed the retrieved papers. Only original articles in English up till 2022 were included. In case of disagreements between researchers when reviewing abstracts, articles were included in the full-text review phase for a final evaluation. The references of all selected articles were also manually reviewed to identify any potentially omitted publications.

This systematic review has been prepared following the PRISMA guidelines.

## 3. Results

### 3.1. Search Results

**Figure 1:**
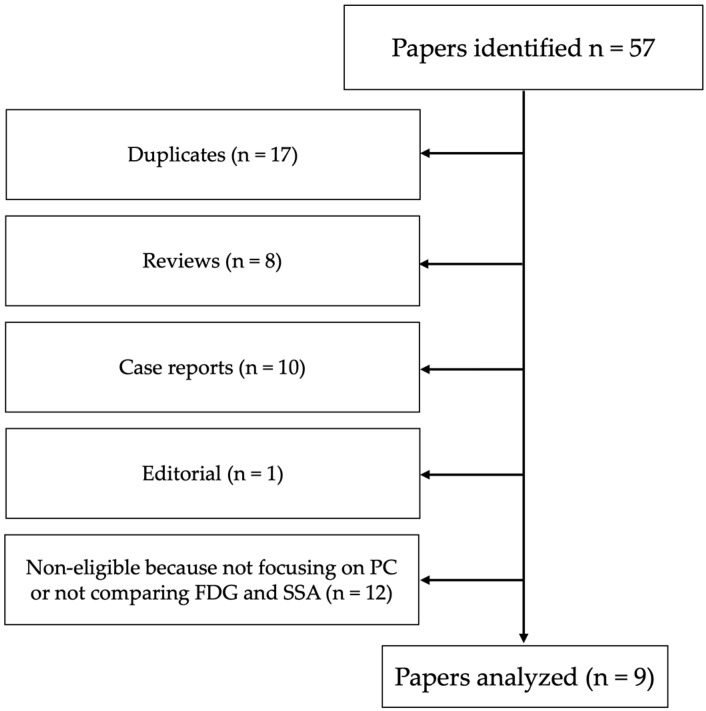
PRISMA flowchart of selected papers.

We found 37 papers in Scopus and 20 in PubMed. Out of these 57 papers, 17 were duplicates, 8 were reviews, 10 were case reports, and 1 was an editorial. Of the remaining 21 papers, 12 were ineligible because they did not focus on PC or did not compare ^68^Ga-SSA and [^18^F]FDG (Figure 1). We carefully read the remaining nine papers, searching for additional papers in their references. 

Results are summarized in Table 1 and Table 2.

### 3.2. Radiological Imaging of Pulmonary Carcinoids

Differential diagnosis of solid pulmonary nodules is always a radiological challenge. Despite being substantially different for histological pattern and a prognosis rarely seen in morphological features, such as regular margins and calcification, permit to discriminate PCs from benign nodules or hamartomas; suspicious imaging features to not overlook in the evaluation of lung nodules, which include interval growth of a solid component that is ≥6 mm, spiculated margins, interval increase in density, or a new microcystic component. Persistent part-solid nodules with solid components ≥6 mm should also be considered highly suspicious. The presence of these features may prompt further diagnostic evaluation.

Several studies showed that TC or RM cannot always characterize solid lung nodules even when intravenous iodate contrast medium is performed [17].

The Fleischner Society recommendations for pulmonary nodules, revised in 2017, suggests that for solitary solid lung nodule >8 mm (>250 mm^3^) CT at 3 months, PET-CT, or tissue sampling should be considered [18].

It is also important to point out that both TCs and ACs show very similar radiological characteristics and for these reasons in most cases morphological exams such as X-ray, contrast enhancement TC or MRI, do not allow to recognize them.

### 3.3. Conventional Somatostatin Receptors Imaging

Somatostatin receptors (SSTR) are over-expressed by neuroendocrine tumors (NENs) [19] and somatostatin receptor scintigraphy (SRS) with ^111^In-pentetreotide (Octreoscan^®^, Mallinckrodt Medical, St. Louis, MO, USA), was first introduced in the 1980s, exploiting the specific binding of a radiolabeled somatostatin analogue (octreotide) to high-affinity somatostatin receptors (mainly type 2) expressed by most neuroendocrine tumors [20]. Thanks to this feature, for many years Octreoscan^®^ has been used in nuclear medicine for localizing, staging and follow-up of tumors with high SSTR expression. Although it has been one of the gold-standard modalities used for the diagnosis of NENs, many studies have shown several limitations, such as unfavorable tumor-to-noise ratio, low spatial resolution, and moderate receptor affinity [21]. For these reasons, research has relied on the use of other radiopharmaceuticals. Two new ^99m^Tc-labelled SST analogs were introduced in the 1990s: [^99m^Tc]Tc-N4-[Tyr3] Octreotate (Demotate^®^, POLATOM, Otwock, Poland) and [^99m^Tc]Tc-EDDA/HYNIC-[Tyr3] Octreotide (Tektrotyd^®^, POLATOM, Otwock, Poland), which have been broadly used in medical practice in patients with several SSTR+ cancers [22,23,24]. With the advent of the new portable ^68^Ge/^68^Ga generators, ^68^Ga-labelled radiopharmaceuticals became available for clinical use with PET/CT. Many are the advantages, and in particular better spatial resolution of PET versus SPECT, shorter scan time and less patient exposure to radiation [25,26].

The introduction of ^68^Ga-SSA PET/CT has indeed improved the diagnostic work-up for the evaluation of lung neuroendocrine tumors which was previously, as mentioned before, only based on traditional imaging such as conventional ultrasound, magnetic resonance imaging (MRI), computed tomography (CT) and somatostatin receptor scintigraphy, becoming one of the gold standard examinations in the diagnosis and management of well-differentiated neuroendocrine tumors [27,28,29]. Three DOTA-peptides (DOTA-TOC, DOTA-NOC and DOTA-TATE) have been purposed in clinical practice for the diagnosis of NENs. DOTA-TATE is also suitable for peptide receptor radionuclide therapy (PRRT) when labelled with ^177^Lutetium [30,31]. The main difference among such compounds is a slightly different affinity for SSR subtypes (mainly type 2, but as well type 3 and type 5), (Table 3). Nevertheless, they have not demonstrated significant diagnostic differences in clinical practice.

Current guidelines recommend PET/CT with ^68^Ga-DOTA-peptides for staging, re-staging, characterization of a bronchial mass and localization of an occult primary neuroendocrine cancer when metastasis of an undetected primary tumor has been demonstrated [19,32]. For ^68^Ga-SSA PET/CT examination, fasting is not required, and it is recommended to have intravenous administration of the labelled compound (dose of 100–200 MBq), with acquisition time varying according to the different conjugated peptide: 45–60 min for ^68^Ga-DOTA-TATE, and 60–90 min for ^68^Ga-DOTA-TOC and ^68^Ga-DOTA-NOC. The amount of labeled peptide injected is less than 50 μg. In the case of patients treated with “cold” somatostatin analogs, appropriate temporary medication withdrawal prior to PET is not strictly necessary. One-day withdrawal is usually prescribed for short-acting somatostatin analogs and three to four weeks for long-acting ones. However, currently, there is no evidence that the interruption of somatostatin analogs administration, prior to PET with ^68^Ga-SSA, is required; it might ameliorate the target-to-background ratios.

^68^Ga-SSA PET/CT, for the staging of neuroendocrine lung tumors, should be routinely performed in addition to conventional imaging techniques, but that alone may not be sufficient to make a correct diagnosis, and it may not identify small distant metastases (especially bone, liver and lymph nodes), [33]. As previously mentioned, PCs express SSTRs on their surface depending on their differentiation. The density of these receptors correlates with the degree of tumor differentiation, with TCs exhibiting the highest density and being the most well-differentiated [19]. For this reason, ^68^Ga-SSA PET/CT offers the possibility to differentiate in vivo histopathological subtypes of PCs (TC and AC) also from poorly differentiated tumors (Large Cell Neuro-Endocrine Carcinoma of the lung, LCNEC and Small Cell Lung Cancer, SCLC). This approach is clinically relevant both for therapeutic and prognostic purposes, since TCs have a much better prognosis with a reported five-year survival of 87–100%, in contrast to ACs, that have a lower, reported five-year survival rate of 25–69% [34]. Moreover, ^68^Ga-SSA PET/CT may also play a key role for patients for whom biopsy, for a variety of reasons, cannot be performed.

In summary, ^68^Ga-SSA PET/CT represents the gold standard imaging modality for the diagnosis and staging of well-differentiated neuroendocrine tumors; in particular, there is solid scientific evidence in GEP/NEN neoplasms [35].

In the last two decades, as reported in the following sections, several studies have evaluated the role of quantitative analysis of ^68^Ga-SSA PET/CT scans for in vivo characterization of PCs (typical versus atypical), adding relevant information on the evaluation of disease extent with the impact of the clinical management of patients [9,36]. A systematic review and meta-analysis regarding 104 patients affected by PCs, published by Jiang et al., reported a higher SUV_max_ on ^68^Ga-SSA PET/CT in TCs as compared with ACs (36.5 versus 9, respectively) [37].

Finally, the European Society of Medical Oncology (ESMO) guidelines, published in 2021, suggested the use of ^88^Ga-SSA PET/CT in addition to contrast-enhanced CT in TNM staging [17].

### 3.4. [^18^F]FDG PET/CT Imaging

[^18^F]FDG represents the main and most widely used radiopharmaceutical for PET imaging, particularly for oncological purposes. [^18^F]FDG is a glucose analog that remains trapped in neoplastic cells depending on their glucose metabolic activity. Its entrance to the cells is mediated by several different Glucose transporters (Glut) expressed on different cell types (Table 4). Some of them are overexpressed in cancer cells [38]. According to the study of Mamede et al., a close correlation between Glut-1 expression and [^18^F]FDG uptake was observed in lung cancer cells [39].

In the management of NENs, however, the role of [^18^F]FDG PET/CT is still a matter of debate, particularly for the evaluation of well differentiated tumors [40,41] but there is solid scientific evidence, that in high-grade tumors there is an upregulation of Glut and glucose metabolism with, often, a loss of SSTR. Indeed, the increase of [^18^F]FDG uptake correlates with the aggressiveness of neoplasia and indicates a worse prognosis [6,42].

In the majority of cases a median activity of 3 MBq/Kg body weight is injected intravenously into euglycemic fasting patients. PET acquisition starts after 60 min [19,29].

As a glucose analog [^18^F]FDG is not specific for tumors, increased activity can also be seen in inflammation or infection by reason of glycolytic activity in leukocytes. Several studies described a high accumulation of [^18^F]FDG in the collapsed lung distal to endobronchial carcinoids secondary to obstructive pneumonia [35,36], whereas ^68^Ga-SSA showed little uptake in the collapsed lung, despite the presence of SSTR on inflammatory cells [43,44].

In addition to visual assessment, [^18^F]FDG uptake can be also quantitatively evaluated. The most used parameters in clinical practice are maximum and mean standardized uptake value (SUV_max_ and SUV_mean_), but metabolic tumor volume (MTV) and total lesion glycolysis (TLG) are also valuable tools in several clinical settings [16].

A study by Jiang et al. [37] showed that the best SUV_max_ cutoff value to distinguish TCs from ACs is 3.7, with 73.9% sensitivity and 65.4% specificity; despite that, the area under the curve of SUV_max_ cutoff value for [^18^F]FDG (AUC: 73.3%) was small and thus making the SUV_max_ value of [^18^F]FDG not a good predictor in differentiation of histological subtype.

Several other studies demonstrated that the SUV_max_ value of [^18^F]FDG might not be reliably used to distinguish more aggressive ACs from less aggressive TCs [36,37]. By contrast, if measured as the ratio between SUV_max_ and SUV_liver_, [^18^F]FDG uptake in PCs correlates with the Ki-67 index being able to identify ACs with a high proliferation index (Ki-67 index of 10–20%) [34,45].

Briganti et al. suggested the use of [^18^F]FDG PET/CT in the follow up of patients: the appearance or an increase of [^18^F]FDG uptake in known lesions would be a sign of worsening related to de-differentiation of the lesion [34].

Recent papers calculated the ratio between SUV_max_ of ^68^Ga-SSA and SUV_max_ of [^18^F]FDG and called it SUVr; this new semi-quantitative parameter is significantly higher in TCs (mean 13.1 ± 7.3, range 1.22 to 30) than ACs [16,37].

Bozkurt et al. recommend the use of [^18^F]FDG for the localization and clinical management of high-grade poorly differentiated NENs with aggressive behavior and as a prognostic factor [32].

Finally, the guidelines of the European Society for Medical Oncology (ESMO, Lugano, Switzerland), published in 2021, suggest the use of [^18^F]FDG in ACs, with high-grade histopathology and when ^68^Ga-SSA PET/CT imaging is negative [17]. Ambrosini et al. in their *Consensus* paper, clearly state that [^18^F]FDG should be used in addition to ^68^Ga-SSA to define in vivo the aggressiveness of the tumor. Moreover, [^18^F]FDG was considered necessary during follow-up, for re-staging in selected patients: (i) if positive at baseline, (ii) in patients with rapid progression of disease despite earlier low-grade disease at histopathology, (iii) for those lesions found by CT/MRI and negative at ^68^Ga-SSA-PET/CT, (iv) as a prognostic factor, and (v) to detect patients with mismatched lesions ([^18^F]FDG positive/^68^Ga-SSA-negative) [29].

### 3.5. Combined ^68^Ga-SSA and [^18^F]FDG PET/CT in Pulmonary Carcinoids

In the last few years, it has emerged that combined imaging with [^18^F]FDG and ^68^Ga-SSA PET/CT for the evaluation of PCs can provide useful information, particularly as a reliable tool in the preoperative assessment of tumor biology. The preoperative distinction between TC and AC is essential both to plan the best surgical strategy and for prognostic purposes. In order to assess the role of combined analysis in the preoperative workup of patients with proven PCs, we systematically analyzed the available literature. We considered only studies regarding PCs in which combined imaging, with both ^68^Ga-SSA PET/CT and [^18^F]FDG PET/CT, was performed (Table 1 and Table 2).

One of the first papers that compared the combined use of ^68^Ga-SSA and [^18^F]FDG in PCs dates back to 2009 [9]. Kayani and coll. evaluated the performance of ^68^Ga-DOTA-TATE and [^18^F]FDG in 18 PCs, of which there were 11 TCs and 2 ACs, and they correlated tumor uptake of each radiopharmaceutical with its metabolic grade at histology. They found a significantly higher uptake of ^68^Ga-DOTA-TATE in all TCs as compared to ACs. [^18^F]FDG uptake was variable in TCs with a SUV_max_ less than 3.4 in about half of patients; in contrast ACs were avid of [^18^F]FDG, with a higher uptake in high grade tumors. Histological grade correlated with the uptake of both radiopharmaceuticals.

Jindal et al. [7] evaluated the distribution of [^18^F]FDG and ^68^Ga-DOTA-TOC in 20 patients with PCs (13 TCs, 7 ACs) to assess the possibility of differentiating the two histopathologic variants based on the different uptake patterns. It was found that the uptake of ^68^Ga-DOTA-TOC was significantly greater in TCs than in ACs (range of SUV_max_ 8.8–66 vs. 1.1–18.5, respectively, *p* < 0.001). Authors also showed that SUV_r_ was significantly higher in TCs than ACs (*p* < 0.001) and they concluded that SUV_r_ is a better predictor of the histopathologic variety of PCs compared with the SUV_max_.

In a prospective study, Venkitaraman and his group [10] compared the diagnostic role of both ^68^Ga-DOTA-TOC PET/TC and [^18^F]FDG PET/TC in patients with suspicious PC, using pathological evaluation (surgery or biopsy) as the gold standard. From 32 patients included in the study 26 resulted in carcinoids (21 TCs and 5 ACs). TCs showed higher ^68^Ga-DOTA-TOC SUV_max_ values than ACs, whereas on the contrary [^18^F]FDG PET SUV_max_ values resulted higher in ACs than in TCs. ^68^Ga-DOTA-TOC performed better than [^18^F]FDG In particular ^68^Ga-DOTA-TOC showed excellent diagnostic ability, with a sensitivity of 96% (100% for TCs and 80% for ACs) and specificity of 100%, while [^18^F]FDG had a sensitivity of 78% (62% for TCs and 100% for ACs) and a specificity of 11%. PPV and NPV for ^68^Ga-DOTA-TOC and [^18^F]FDG were 100% and 86% vs. 69% and 17%, respectively, with an accuracy of 97% vs. 59%.

Lococo et al. [11] in their multicenter study retrospectively evaluated the detection rate (DR) of both ^68^Ga-DOTA-peptide and [^18^F]FDG performed in 33 patients with pathologically confirmed pulmonary carcinoid (23 TCs and 10 ACs). Overall, ^68^Ga-DOTA-peptide was positive in 26 cases (DR 79%), while [^18^F]FDG was positive in 18 cases (DR 55%). Regarding histologic subtypes, ^68^Ga-DOTA-peptide PET/CT was significantly superior to [^18^F]FDG in detecting TCs (91% vs. 35%), while [^18^F]FDG PET/CT was significantly better in detecting AC (100% vs. 50%). Authors also proposed, in order to predict the histological diagnosis, other semiquantitative parameters beyond SUV_max_, such as SUV_T/L_ (target-to-liver ratio) for [^18^F]FDG, SUV_T/S_ (target-to-spleen ratio) for ^68^Ga-DOTA-peptides, and SUV_r_ (SUV_max_ of ^68^Ga-DOTA-peptides divided by SUV_max_ of [^18^F]FDG). [^18^F]FDG SUV_max_ and SUV_T/L_ resulted significantly higher in ACs, whereas SUV_T/S_, ^68^Ga-DOTA-peptides SUV_max_ and SUV_r_ were significantly higher in TCs, and a cutoff value of 1.19 for this latter parameter was accurate to distinguish TCs from ACs (sensitivity 82.6%; specificity 90%).

The same group some years later [12] retrospectively confirmed the utility of both radiopharmaceuticals for the assessment of PCs in a larger monocentric cohort of patients (*n* = 62, 55 TCs and 7 ACs) that underwent [^18^F]FDG and/or ^68^Ga-DOTA-TOC followed by surgical resection. Twenty-six patients underwent only ^68^Ga-DOTA-TOC, fifty-two patients only [^18^F]FDG and twenty patients performed both examinations. Overall, the diagnostic performance of ^68^Ga-DOTA-TOC was superior compared to [^18^F]FDG; in particular, by using a SUV_max_ threshold of 2.5, the DR was 88.4% vs. 53.8%, respectively, and was even more remarkable using a SUV_max_ threshold of 1.5, where the DR reached 100% vs. 80.8%. Nevertheless, considering both SUV_max_ thresholds, ^68^Ga-DOTA-TOC performed better only in TCs, while in ACs, the [^18^F]FDG diagnostic ability was superior. PET results also correlated with histopathological features; in particular ^68^Ga-DOTA-TOC positivity showed a significant correlation with a low mitotic rate, while ^68^Ga-DOTA-TOC negativity seemed to be associated with presence of necrosis.

Komek et al. [13] also evaluated the diagnostic performance of the combined use of [^18^F]FDG and ^68^Ga-DOTA-TATE PET/TC in patients with proven diagnoses of PC. Among 20 patients retrospectively included in the study, 17 underwent surgery, while 3 were only biopsies; 13 resulted in TC and 7 AC. Only 3 cases, all atypical PC had lymph node involvement, and one of those also presented bone metastasis. DR for TC was 100% with ^68^Ga-DOTA-TATE, while [^18^F]FDG detected 11 of 13 cases (84%). In ACs, DR was 100% for both. [^18^F]FDG showed significantly higher SUV_max_ values in ACs, while ^68^Ga-DOTA-TATE showed significantly higher SUV_max_ values in TC. The authors also found a negative correlation between [^18^F]FDG and ^68^Ga-DOTA-TATE SUV_max_ values, suggesting that those findings can predict in vivo the histological subtype.

Zidan et al. [14] in their retrospective work also analyzed the combined use of [^18^F]FDG and ^68^Ga-DOTA-TATE PET/TC in 56 patients with histological diagnosis of well differentiated PC (22 TCs and 34 ACs), focusing in particular on their role for a selection of patients suitable for peptide receptor radionuclide therapy (PRRT). [^18^F]FDG avidity was assessed comparing tumor-to-liver uptake, with a Deauville Score-like scale: 0, no uptake; 1, above blood-pool and less than liver; 2, equal to the liver; 3, moderately above the liver; 4, markedly above the liver. Scores 3 and 4 were considered as [^18^F]FDG positive, 0–2 as negative. ^68^Ga-DOTA-TATE avidity was graded based on the modified Krenning scale: 0, no uptake; 1, less than the liver; 2, equal to the liver; 3, equal to the liver but less than the spleen; 4, equal or more than the spleen or SUV_max_ ≥ 30 in absence of the spleen. Thus, patients were grouped in four different molecular imaging (MI) phenotype: 1, lesions negative on both tracers; 2, lesions ^68^Ga-DOTA-TATE positive and [^18^F]FDG negative; 3, lesions positive on both tracers; 4, lesions [^18^F]FDG positive and ^68^Ga-DOTA-TATE negative. Scores 2 and 3 (50% of patients) were considered suitable for PRRT. Overall, 34% of patients showed tumor heterogeneity having more than one MI phenotypes, regardless of the histological subtype, with similar proportions of TCs and ACs. In the subgroup of 16 patients that finally underwent PRRT, excluding one case with a score 4 that was treated despite being unsuitable for therapy for uncontrolled symptoms and palliative debulking of the disease that had PD, the DR was 85% (46% PR and 39% SD), highlighting the importance of this dual-tracer approach to identify patients that can benefit the most from this therapy.

Deleu et al. [15] recently found in a cohort of 64 patients with pathological diagnosis of PC (52 TCs and 12 ACs), higher ^68^Ga-SSA SUV_max_ in TCs and higher [^18^F]FDG SUV_max_ in ACs. For both techniques, SUV_max_ values were significantly different between TCs and ACs. Furthermore, the authors also aimed to assess the ability of ^68^Ga-SSA PET/CT to detect lymph nodes and distant metastases relying on pathological specimens. In a total of 267 hylo-mediastinal lymph node stations from 56 patients that underwent dissection or biopsy, the sensitivity and the specificity for regional lymph node involvement by ^68^Ga-SSA PET/CT was 80% and 75%, respectively, applying a SUV_max_ cutoff value of 2.1, with the true positive rate that topped to 100% using a SUV_max_ cutoff value of 4.0. Moreover, all 12 lesions from 10 patients were seen by ^68^Ga-SSA PET/CT as suspicious for distant metastases and afterward biopsied confirmed to be metastatic (PPV of 100%).

An interesting recent multicentric study [16] investigated the use of ^68^Ga-DOTA-TATE and [^18^F]FDG, in 61 patients with histologically confirmed lung carcinoids, for predicting the nature of pulmonary carcinoids (typical vs. atypical) and for the evaluation of their prognostic role. PET images were visually analyzed and SUV_max_ of the lung lesion was calculated for semiquantitative analysis. SUV_max_ was also calculated for the spleen, liver, and blood and divided by SUV_max_ of the lung lesion in each patient to obtain lesion-to-spleen SUV ratio (L-S SUVr), lesion-to-liver SUV ratio (L-L SUVr), and lesion-to-blood-pool SUV ratio (L-BP SUVr). Using both radiopharmaceuticals, PET examination were classified as follows: score 1 [^18^F]FDG and ^68^Ga-SSA negative; score 2, ^68^Ga-SSA positive and [^18^F]FDG negative; score 3, ^68^Ga-SSA negative and [^18^F]FDG positive; score 4, both PET/CT positive. Authors also calculated, for each patient, the ratio of SUV_max_ on ^68^Ga-SSA over that on [^18^F]FDG (SUVr). This retrospective study showed that dual radiopharmaceutical approach provides complementary information about different biological features of PCs, and thus helping in the prediction of histological diagnosis. Authors also suggested in distinguishing TCs and ACs both qualitative and semiquantitative analysis; in particular, the SUVr whose best value to predict the final diagnosis was 1.05 (AUC 0.889). Finally, the results of this study demonstrated also their prognostic role in predicting PFS. Only histological subtypes were correlated both with PFS and OS at a multivariate analysis and among PET/CT features, only [^18^F]FDG and ^68^Ga-SSA were independently related to PFS. Concerning SUVr, this variable is significantly related to the OS only at univariate analysis but may give useful information in a presurgical field where an exact histological diagnosis is not yet known.

## 4. Conclusions

**Figure 2:**
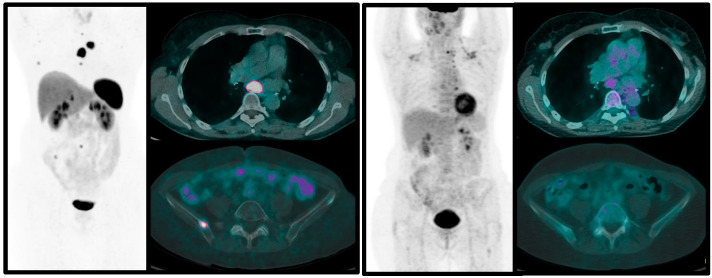
Sixty-three-year-old female patient with Atypical PC (Ki-67 20%). The combined imaging with [^18^F]FDG (**left**) and ^68^Ga-DOTA-NOC PET/CT (**right**) show high glucose metabolism of the lesions and faint somatostatin receptor expression, suggesting an aggressive histotype.

**Figure 3:**
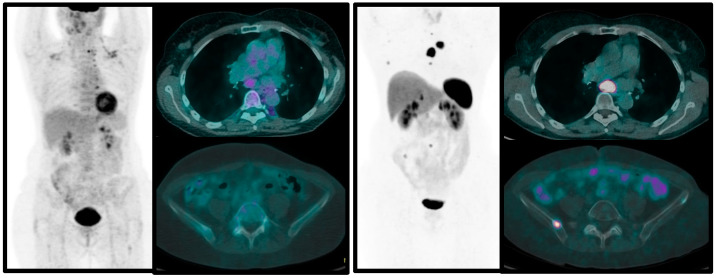
Fifty-eight-year-old female patient with previous history of TC of the inferior left lobe, treated with lobectomy, that 20 years after surgery developed multiple bone and nodal metastases (confirmed by biopsy). The patient underwent [^18^F]FDG (**left**) and ^68^Ga-DOTA-TOC PET/TC (**right**) within one week. All lesions show very high somatostatin receptors expression, but low glucose metabolism.

From the few published papers, it emerges that PET/CT with ^68^Ga-labeled SSA should always be performed in patients with suspicion of pulmonary NENs for staging purposes and to decide therapeutic options. [^18^F]FDG PET/CT is indicated in atypical carcinoids and in typical carcinoids during the follow-up, when progression of the disease is known or suspected, to define the biological switch to a more aggressive form (Figure 2 and Figure 3). The combined approach with these two functional imaging modalities in clinical practice enables more accurate staging, definition of aggressiveness and tumor biology, with a prognostic role. Nevertheless, more data should be published on homogeneous series of patients in order to evaluate the real impact of dual imaging on overall survival and progression-free rate of this disease.

## Figures and Tables

**Table 1:** Characteristics of selected publications.

Reference	Patients	Research Type	Histology	Radiopharmaceuticals
Kayani I et al. [9]	13	Retrospective	11 TC, 2 AC	^68^Ga-DOTA-TATE and [^18^F]FDG
Jindal T et al. [7]	20	Retrospective	13 TC, 7 AC	^68^Ga-DOTA-TOC and [^18^F]FDG
Venkitaraman B et al. [10]	26	Prospective	21 TC, 5 AC	^68^Ga-DOTA-TOC and [^18^F]FDG
Lococo F et al. [11]	33	Retrospective	23 TC, 10 AC	^68^Ga-DOTA-TATE/NOC/TOC and [^18^F]FDG
Lococo F et al. [12]	62	Retrospective	55 TC, 7 AC	^68^Ga-DOTA-TOC and [^18^F]FDG
Komek H et al. [13]	20	Retrospective	13 TC, 7 AC	^68^Ga-DOTA-TATE and [^18^F]FDG
Zidan L et al. [14]	56	Retrospective	22 TC, 34 AC	^68^Ga-DOTA-TATE and [^18^F]FDG
Deleu AL et al. [15]	64	Retrospective	52 TC, 12 AC	^68^Ga-DOTA-TATE/TOC and [^18^F]FDG
Albano D et al. [16]	61	Retrospective	35 TC, 26 AC	^68^Ga-DOTA-TATE and [^18^F]FDG

**Table 2:** Summary of the 9 publications on pulmonary carcinoids studied with both ^68^Ga-SSA and [^18^F]FDG.

Title	Comments and Conclusions	Reference
A comparison of ^68^Ga-DOTA-TATE and [^18^F]FDG in pulmonary neuroendocrine tumors	Role of combined imaging with ^68^Ga-DOTA-TATE and [^18^F]FDG in PCs, correlating uptake values with metabolic grade and histology (TCs vs. ACs)	Kayani I et al. [9]
Evaluation of the role of [^18^F]FDG and ^68^Ga-DOTA-TOC in differentiating typical and atypical pulmonary carcinoids	The analysis of tumor uptake parameters on [^18^F]FDG and ^68^Ga-DOTA-TOC was able to differentiate between typical and atypical carcinoids	Baudin E et al. [7]
The diagnostic and prognostic role of combined [^18^F]FDG and ^68^Ga-DOTA-peptides in primary pulmonary carcinoids: a multicentric experience	Importance of dual tracer approach with ^68^Ga-DOTA-TATE and [^18^F]FDG to stratify patients in order to predict histological results (TC or AC) and give prognostic information	Albano D et al. [16]
Multicenter comparison of [^18^F]FDG and ^68^Ga-DOTA-peptide for pulmonary carcinoid	DR evaluation of ^68^Ga-DOTA-peptide and [^18^F]FDG in PC, with proposal of some semi-quantitative parameters to distinguish between TCs and ACs	Lococo F et al. [11]
Diagnostic performances of ^68^Ga-DOTA-TOC versus ^18^Fluorodeoxyglucose positron emission tomography in pulmonary carcinoid tumors and interrelationship with histological features	Diagnostic ability of [^18^F]FDG and ^68^Ga-DOTA-TOC in PCs and correlation of the results with histopathology after surgery	Lococo F et al. [12]
Comparison of [^18^F]FDG and ^68^Ga-DOTA-TATE imaging methods in terms of detection of histological subtype and related SUVmax values in patients with pulmonary carcinoid tumors	Combined use of ^1^[^18^F]FDG and ^68^Ga-DOTA-TATE in patients with PCs; DR analysis with a focus on histological prediction (TC vs. AC)	Komek H et al. [13]
Value of ^68^Ga-somatostatin in the grading of pulmonary neuroendocrine (carcinoid) tumors and the detection of disseminated disease: single-center pathology-based analysis and review of the literature	Importance of dual tracer approach to differentiate between ACs and TCs, highlighting the excellent performance of SSTR PET/CT to detect nodal and distant metastases confirmed by pathological analysis	Deleu AL et al. [15]
Role of ^68^Ga-DOTA-TOC in initial evaluation of patients with suspected bronchopulmonary carcinoid	Showing the superior diagnostic performance of ^68^Ga-DOTA-TOC compared to [^18^F]FDG in patients with suspect PC, and correlation with histopathology	Venkitaraman B et al. [10]
Theranostic implications of molecular imaging phenotype of well-differentiated pulmonary carcinoid based on ^68^Ga-DOTA-TATE and [^18^F]FDG	Introducing some scores to define tumor heterogeneity thanks to the combined approach with [^18^F]FDG and ^68^Ga-DOTA-TATE, in order to have the optimal selection of patients with PC suitable for PRRT	Zidan L et al. [14]

**Table 3:** Cell expression and up-regulated expression of somatostatin receptors.

SSTR	Positive Human Tumors	Cell Line	Affinity for ^68^Ga-SSA
SSTR 1	Breast carcinoma, Paragangliomas, Prostate cancer, Sarcomas, Inactive pituitary adenomas, GEP-NET, Pheochromocytomas, Gastric cancer, and Ependymomas	Colon cancer (with neuroendocrine features), and Gastric cancer	Low
SSTR 2	Neuroblastomas, Meningiomas, Medulloblastomas, Breast cancer, Lymphomas, Renal cell carcinomas, Paragangliomas, Small cell lung cancer, Hepatomas Sarcomas, Inactive pituitary adenomas, GH-secreting adenomas, GEP-NET, Pheochromocytomas, and Gastric cancer	Breast cancer, Colon cancer, Gastric cancer, and Glioblastoma	Very high
SSTR 3	Paragangliomas (low density), Inactive pituitary adenomas, GH-secreting adenomas (low density), and GEP-NET	Lung cancer (squamous), and Colon cancer (with neuroendocrine features)	High
SSTR 4	Sarcomas	Monoblastic leukemia, and Breast cancer	Low
SSTR 5	Lymphomas (low density), Prostate cancer (low density), Inactive pituitary adenomas, GH-secreting adenomas, GEP-NET, Pheochromocytomas (low density), Gastric cancer, and Ependymomas	Breast cancer, Gastric cancer, and Colon cancer	High

**Table 4:** Cell expression and up-regulated expression of glucose transporters.

GLUT	Tissue Distribution	Sub-Cellular Localization	Substrate Selectivity	Up-Regulated Expression
GLUT 1	Widely distributed, and highly expressed especially in erythrocytes, brain, testis and kidney	Cell membrane	Glucose, dehydriascorbic acid	Tumor cells, endothelial cells, renal cells, skeletal muscle cells, adipocytes, hepatocytes, and brain
GLUT 2	Widely distributed, and highly expressed in erythrocytes, brain, testis and kidney	Cell membrane	Glucose, and fructose	Unclear
GLUT 3	Widely distributed, and highly expressed in erythrocytes, brain, testis and kidney	Cell membrane	Glucose	Tumor cell, and pancreatic cells
GLUT 4	Adipose tissue, skeletal, and cardiac muscle	Cell membrane	Insulin	Skeletal muscle cells
GLUT 5	Small intestine, kidney, testis, and adipose cell	Cell membrane	Fructose	Tumor cells
GLUT 6	Brain, spleen, immune cells, and adipose cell	Cell membrane	Glucose	Brain
GLUT 7	Small intestine and colon, testis and prostate	Cell membrane	Glucose and fructose	Small intestine tissue
GLUT 8	Brain (hippocampus), testis, epencephalon, and adrenal glands	Cell membrane, and endoplasmic reticulum	Glucose, fructose and trehalose	Neuronal cells and tumor cells
GLUT 9	Liver, kidneys, small intestine, leukocytes, and chondrocytes	Cell membrane	Uric acid	Ovarian granular cells
GLUT 10	Liver and pancreas	Cell membrane, endoplasmic reticulum	Glucose	Brain tissue
GLUT 11	Skeletal muscles, heart, kidneys, adipose tissue, placenta, and pancreas	Cell membrane	Glucose	Unclear
GLUT 12	Skeletal muscles, heart, placenta, and prostate	Cell membrane and intracellular organelles	Glucose, fructose and galactose	Mammary tumor cells
GLUT 13	Cerebrum, adipose tissue, and kidneys	Cell membrane and vesicles	Inositol	Unclear
GLUT 14	Testis	Cell membrane	Unclear	Unclear

## Data Availability

Not applicable.
